# Predictive value of the systemic immune inflammation (SII) index for stroke‐associated pneumonia

**DOI:** 10.1002/brb3.3302

**Published:** 2023-11-08

**Authors:** Zhanhang Cui, Sai Kuang, Xiaorong Yang, Ying Wang, Shanshan Gu, Hongyu Li, Huibin Chen, Yanbing Han, Haimei Sun

**Affiliations:** ^1^ School of Public Health Kunming Medical University Kunming, Yunnan Province China; ^2^ Department of Critical Care Medicine First Affiliated Hospital of Kunming Medical University Kunming, Yunnan Province China; ^3^ Department of Neurology First Affiliated Hospital of Kunming Medical University Kunming, Yunnan Province China

**Keywords:** acute stroke, predictive value, stroke‐associated pneumonia, systemic immune inflammation index

## Abstract

**Objective:**

To investigate the predictive value of the systemic immune inflammation (SII) index on the occurrence of stroke‐associated pneumonia (SAP) in patients with acute stroke.

**Methods:**

Data of patients with or without a previous history of pulmonary who visited the First Affiliated Hospital of Kunming Medical University within 24 h of the onset of stroke were collected between January 2017 and December 2019. Patient's demographic data, stroke type, past medical history, National Institutes of Health Stroke Scale score, Glasgow Coma score, and laboratory tests were collected. Logistic regression models and receiver‐operating characteristic (ROC) curves were used to investigate the predictive value of SII for the development of SAP in patients with stroke.

**Results:**

We included 395 patients with acute stroke, with a mean age of 63.89 ± 13.42 years, of whom 340 (86.1%) had ischemic stroke, and 55 (13.9%) had hemorrhagic stroke. Out of 395, 113 (28.6%) had SAP and 282 (71.4%) did not, and the SII level in the SAP group was higher than that of the non‐SAP group (*p* < .05). Logistic regression analysis of patients with stroke showed that higher SII was a risk factor for SAP in patients with stroke (per 100 units, HR = 1.081, 95% CI: 1.035–1.130, *p* < .001), and tertile grouping of SII showed that the risk of SAP was 5.059 times higher in the SIIQ3 group than in the SIIQ1 group (95% CI: 2.061–12.418, *p* < .001). ROC curve analysis indicated that the SII index had predictive value for the occurrence of SAP in patients with stroke, with an area under the curve of 0.752 (95% CI: 0.698–0.806). When the cutoff value was 861.01, the SII predicted SAP in patients with stroke with a sensitivity of 61.9% and a specificity of 76.2%.

**Conclusion:**

Higher SII is an independent risk factor for the development of SAP in patients with stroke and has some predictive value for the development of SAP.

## Graphical abstract

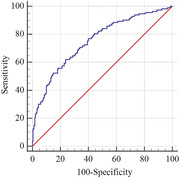

## 1 INTRODUCTION

Stroke is a type of disease that results from brain damage due to insufficient blood supply to the brain caused by rupture (hemorrhagic stroke) or blockage (ischemic stroke) of blood vessels in the brain and is a common cause of death (Liu et al., 2019; Zhou et al., 2016). The mortality rate of patients with stroke is currently decreasing (He et al., 2014; Li et al., 2016). However, the clinical outcomes of stroke are of widespread concern. Stroke‐associated pneumonia (SAP) is one of the most common complications in patients with stroke. It may be due to a combination of poststroke aspiration and changes in immune function (Hoffmann et al., 2017). Aspiration can lead to infections from pathogenic bacteria in patients with stroke, whereas immune suppression creates an environment conducive to the pathogens. SAP increases the length of hospital stay, financial burden, and mortality rate of patients with stroke (Katzan et al., 2007; Suda et al., 2018; Vermeij et al., 2009; Wilson, 2012; Yu et al., 2016). Therefore, accurate and rapid prediction of SAP is crucial in patients with stroke.

The systemic immune inflammation (SII) is one of the most commonly used prognostic indicators of inflammation. It can assess body inflammation and immune function in various cancers, such as esophageal squamous cell carcinoma and gastric cancer (Inoue et al., 2021; Luo et al., 2019; Yang et al., 2018; Ye et al., 2022). Zhao et al. (2020) reported that in patients with stroke, immunosuppression and inflammatory responses decreased the number of lymphocytes and platelets while increasing the number of neutrophils. Kim et al. reported that the SII index, which is based on three cell types, provides a more comprehensive picture of patients’ immune and inflammatory responses (Zhao et al., 2020) and is also independently associated with stroke severity at the time of patient admission (Hou et al., 2021). Therefore, we hypothesized that SII, an inflammatory marker composed of neutrophil, lymphocyte, and platelet counts, could predict SAP. Thus, this study aimed to investigate the diagnostic value of SII in predicting the occurrence of SAP in patients with acute stroke.

## 2 PATIENTS AND METHODS

### 2.1 Study population

In this study, we included 395 patients admitted to the First Affiliated Hospital of Kunming Medical University within 24 h of the onset of acute stroke between January 2017 and December 2019. The inclusion criteria were as follows: (i) above 18 years of age; (ii) diagnosis of acute stroke confirmed by computed tomography or magnetic resonance imaging of the brain within 48 h of admission; (iii) patients with or not a previous history of pulmonary; (iv) complete clinical data.

Exclusion criteria were as follows: (i) infectious or inflammatory diseases prior to admission; (ii) concomitant hematological disorders or tumors; (iii) transient cerebral ischemia; and (iv) use of long‐term immunosuppressive drugs.

### 2.2 Data collection

Within 24 h of patient admission, demographic, clinical, and laboratory data were collected, including age, sex, body mass index, stroke type, previous stroke history, smoking, alcohol consumption, heart attack, atrial fibrillation, coronary artery disease, use of acid‐suppressing medications, presence of indwelling gastric tube, National Institutes of Health Stroke Scale (NIHSS) score (Kwah & Diong, 2014), Glasgow Coma score (Jain & Iverson, 2022), absolute neutrophil count, absolute lymphocyte count, and platelet count (SII index = absolute neutrophil count × platelet count/absolute lymphocyte count).

### 2.3 Statistical analysis

SPSS 24.0 software (IBM Inc) was used for statistical analysis; the measured data were tested for normality using Shapiro–Wilk. Data following normal distribution was expressed as mean ± standard deviation ($\bar{x}$ ± *s*), and *t*‐test was used to compare the two groups. Data that did not follow a normal distribution were expressed as median (P25, P75). Categorical data were expressed as frequencies (composition ratio), and the *χ* ^２^ test was used to compare categorical data between group. Logistic regression analysis was used to examine the risk factors for SAP in patients with stroke. The SII index was classified into lower quantile Q1, middle quantile Q2, and higher quantile Q3 based on tertiles. The trend relationship between SII and the risk of SAP in patients with stroke was examined. The receiver‐operating characteristic (ROC) curve was used to evaluate the predictive value of SII for concurrent SAP in patients with stroke. *p* < .05 was considered to be statistically significant.

## 3 RESULTS

### 3.1 Comparison of general and clinical data between non‐SAP and SAP groups

Data were collected from 450 patients with acute stroke, of whom 395 (88%) were eligible patients with a mean age of 63.89 ± 13.42 years. Out Of them, 340 (86.1%) had ischemic stroke, and 55 (13.9%) had hemorrhagic stroke. Of the eligible patients, 250 (63.3%) were men, and 145 (36.7%) were women. The SAP group consisted of 113 (28.6%) patients, including 68 (60.2%) men and 45 (39.8%) women. There were 282 (71.4%) non‐SAP patients, including 182 (64.5%) men and 100 (35.5%) women. Table 1 shows the results of analysis performed between the groups showing that the percentage of age, NIHSS score, presence of heart failure, use of an indwelling gastric tube, neutrophil count, mortality during hospitalization, and SII index were higher in the SAP group than those in the non‐SAP group (*p* < .05).

**TABLE 1:** Comparison of general and clinical data between non‐stroke‐associated pneumonia (SAP) and SAP groups.

Variables	Overall (*n* = 395)	Non‐SAP group (*n* = 282)	SAP group (*n* = 113)	*t*/*z*/*x* ^2^ Value	*p*‐Value
Age, years, mean ± SD	63.89 ± 13.42	62.19 ± 13.28	68.12 ± 12.87	4.051	<.001
Male, *n* (%)	250 (63.3)	182 (64.5)	68 (60.2)	0.661	.416
BMI, kg/m^2^	23.79 ± 3.66	24.08 ± 3.65	23.09 ± 3.60	−2.444	.015
Stroke type				2.868	.090
Ischemic stroke, *n* (%)	340 (86.1)	248 (87.9)	92 (81.4)		
Hemorrhagic stroke, *n* (%)	55 (13.9)	34 (12.1)	21 (18.6)		
Subtentorial, *n* (%)	78 (19.8)	57 (20.2)	21 (18.6)	0.135	.713
Supratentorial, *n* (%)	286 (72.4)	196 (69.5)	90 (79.6)	4.154	.042
COPD, *n* (%)	11 (2.8)	5 (1.8)	6 (5.3)	2.535	.111
Myocardial infarction, *n* (%)	7 (1.8)	5 (1.8)	2 (1.8)	0.000	1.000
Atrial fibrillation, *n* (%)	32 (8.1)	18 (6.4)	14 (12.4)	3.909	.048
Coronary heart disease, *n* (%)	37 (9.4)	27 (9.6)	10 (8.8)	0.050	.823
Diabetes mellitus, *n* (%)	75 (19.0)	53 (18.8)	22 (19.5)	0.024	.877
Stroke history, *n* (%)	74 (18.7)	53 (18.8)	21 (18.6)	0.002	.961
Hypertension, *n* (%)	263 (66.6)	190 (67.4)	73 (64.6)	0.279	.597
Hyperlipemia, *n* (%)	27 (6.8)	18 (6.4)	9 (8.0)	0.317	.573
Heart failure, *n* (%)	18 (4.6)	7 (2.5)	11 (9.7)	9.756	.002
Gout, *n* (%)	12 (3.0)	10 (3.5)	2 (1.8)	0.366	.545
Current smoker, *n* (%)	201 (50.9)	144 (51.1)	57 (50.4)	0.012	.911
Current drinker, *n* (%)	187 (47.3)	133 (47.2)	54 (47.8)	0.013	.911
Gastric intubation, *n* (%)	86 (21.8)	21 (7.4)	65 (57.5)	118.772	<.001
Acid suppression drugs, *n* (%)	269 (68.1)	169 (59.9)	100 (88.5)	30.305	<.001
Water swallow test [IQR]	1.00 (1.00,1.00)	1.00 (1.00,1.00)	1.00 (1.00,3.50)	−7.126	<.001
Glucose value [IQR]	5.83 (4.90,7.30)	5.50 (4.70,6.60)	6.60 (5.60,8.55)	−5.404	<.001
Neutrophil counts (×10^9^/L)	4.72 (3.57,7.34)	4.39 (3.41,5.63)	7.58 (4.67,10.45)	−7.803	<.001
Lymphocyte counts (×10^9^/L)	1.61 (1.20,2.02)	1.75 (1.38,2.08)	1.20 (0.80,1.57)	−7.635	<.001
Platelet counts (×10^9^/L)	198.00 (161.00,241.00)	201.00 (163.75,250.25)	189.00 (153.50–222.50)	−2.535	.011
In‐hospital mortality, *n* (%)	10 (2.5)	1 (0.4)	9 (8)	15.975	<.001
NIHSS at admission [IQR]	4.00 (2.00,9.00)	3.00 (2.00,6.00)	11.00 (8.00,14.00)	−9.525	<.001
GCS at admission [IQR]	15.00 (13.00,15.00)	15.00 (14.00,15.00)	11.00 (8.00,14.00)	−10.786	<.001
SII at admission [IQR]	624.18 (369.45,1051.03)	509.42 (320.78,844.65)	1087.45 (624.30,1989.97)	−7.844	<.001

Abbreviations: BMI, body mass index; COPD, chronic obstructive pulmonary disease; GCS, Glasgow Coma score; IQR, interquartile range; NIHSS, National Institutes of Health Stroke Scale; SII, systemic immune inflammation index.

### 3.2 Logistic regression analysis of the relationship between SII and SAP in patients with stroke

The occurrence of SAP was used as the dependent variable, and SII as the independent variable in a multifactorial logistic analysis. Table 2 shows that older age (HR = 1.040, 95% CI: 1.012–1.069, *p* = .005), concomitant heart failure (HR = 5.260, 95% CI: 1.439–19.221, *p* = .012), use of acid‐suppressing medications (PPI) (HR = 2.475, 95% CI: 1.140–5.370, *p* = .022), use of the indwelling gastric tube (HR = 4.753, 95% CI: 2.028–11.138, *p* < .001), NIHSS score (HR = 1.090, 95% CI: 1.009–1.177, *p* = .028), and higher SII (per 100 units, HR = 1.081, 95% CI: 1.035–1.130, *p* < .001) were risk factors for the development of SAP in patients with stroke.

**TABLE 2:** Dichotomous logistic regression analysis of factors influencing stroke‐associated pneumonia (SAP).

			Model 1		Model 2		Model 3	
Features	Patients at risk, *N*	Events, *N*	HR (95%CI)	*p*‐Value	HR (95%CI)	*p*‐Value	HR (95%CI)	*p*‐Value
Age, years	395	113	1.041 (1.020–1.062)	<.001	1.038 (1.012–1.066)	.005	1.040 (1.012–1.069)	.005
Gender								
Women	145	45						
Male	250	68	0.970 (0.580–1.622)	.908	0.644 (0.2656–1.562)	.330	0.749 (0.301–1.863)	.534
BMI, kg/m^2^	395	113	0.965 (0.899–1.035)	.320	1.006 (0.918–1.102)	.898	0.994 (0.903–1.095)	.904
Subtentorial								
No	317	92						
Yes	78	21			3.129 (0.735–13.312)	.123	4.331 (0.879–21.352)	.072
Supratentorial								
No	109	23						
Yes	286	90			5.071 (1.359–18.920)	.016	5.699 (1.290–25.166)	.022
COPD								
No	384	107						
Yes	11	6			2.416 (0.474–12.336)	.288	2.321 (0.343–15.717)	.388
Myocardial infarction								
No	388	111						
Yes	7	2			0.690 (0.036–13.336)	.806	0.661 (0.028–15.435)	.797
Atrial fibrillation								
No	363	99						
Yes	32	14			0.909 (0.300–2.750)	.865	0.586 (0.168–2.050)	.403
Coronary heart disease								
No	358	103						
Yes	37	10			0.654 (0.208–2.051)	.466	0.825 (0.250–2.727)	.753
Diabetes mellitus								
No	320	91						
Yes	75	22			1.059 (0.471–2.381)	.889	1.310 (0.573–2.995)	.522
Stroke history								
No	321	92						
Yes	74	21			0.890 (0.410–1.933)	.768	0.705 (0.301–1.651)	.421
Hypertension								
No	132	40						
Yes	263	73			0.617 (0.306–1.245)	.178	0.803 (0.375–1.717)	.571
Hyperlipemia								
No	368	104						
Yes	27	9			0.976 (0.286–3.324)	.969	0.927 (0.264–3.256)	.906
Heart failure								
No	377	102						
Yes	18	11			5.365 (1.425–20.195)	.013	5.260 (1.439–19.221)	.012
Gout								
No	383	111						
Yes	12	2			0.658 (0.094–4.607)	.673	0.794 (0.115–5.490)	.815
Current smoker								
No	194	56						
Yes	201	57			1.132 (0.273–4.691)	.864	0.807 (0.192–3.394)	.770
Current drinker								
No	208	59						
Yes	187	54			1.311 (0.341–5.037)	.693	1.596 (0.410–6.205)	.500
Gastric intubation								
No	309	48						
Yes	86	65			11.600 (5.807–23.174)	<.001	4.753 (2.028–11.138)	<.001
Acid‐suppressing drugs								
No	126	13						
Yes	269	100			2.941 (1.381–6.266)	.005	2.475 (1.140–5.370)	.022
Water swallow test	395	113					1.068 (0.772–1.478)	.689
Glucose value	395	113					0.969 (0.918–1.022)	.250
NIHSS at admission	395	113					1.090 (1.009–1.177)	.028
GCS at admission	395	113					0.842 (0.711–0.998)	.048
Per 100 units SII at admission	395	113	1.135 (1.094–1.177)	<.001	1.103 (1.059–1.149)	<.001	1.081 (1.035–1.130)	<.001

*Note*: Model 1: corrected for age, gender, BMI. Model 2: corrected for age, gender, BMI, subtentorial, supratentorial, COPD, myocardial infarction, atrial fibrillation, coronary heart disease, diabetes mellitus stroke history, hypertension, hyperlipemia, heart failure, gout, current smoker, current drinker, gastric intubation, acid‐suppressing drugs. Model 3: corrected for age, gender, BMI, subtentorial, supratentorial, COPD, myocardial infarction, atrial fibrillation, coronary heart disease, diabetes mellitus, stroke history, hypertension, hyperlipemia, heart failure, gout, current smoker, current drinker, gastric intubation, acid‐suppressing drugs, water swallow test, glucose, NIHSS, GCS.

Abbreviations: COPD, chronic obstructive pulmonary disease; GCS, Glasgow Coma score; NIHSS, National Institutes of Health Stroke Scale; SII, systemic immune inflammation.

### 3.3 Trends in the association between SII and the development of SAP in patients with stroke

Table 3 shows the results of analysis after grouping study participants according to SII tertiles, revealing that the risk of SAP was 9.356 times higher in Q3 group than in Q1 group without correction for confounders (95% CI: 4.803–18.224, *p* < .001). The risk of developing SAP was 5.059 times (95% CI: 2.061–12.418, *p* < .001) higher in Q3 group than in Q1 group after correction for confounders.

**TABLE 3:** Logistic regression model of the systemic immune inflammation (SII) index and the risk of developing stroke‐associated pneumonia (SAP) in patients.

	Events	Model 1		Model 2		Model 3		Model 4	
	*n* (%)	HR (95%CI)	*p*‐Value	HR (95%CI)	*p*‐Value	HR (95%CI)	*p*‐Value	HR (95%CI)	*p*‐Value
Q1	13 (9.9)								
Q2	33 (25.0)	3.026 (1.510–6.064)	.002	3.057 (1.505–6.210)	.002	4.096 (1.724–9.734)	.001	3.289 (1.333–8.111)	.010
Q3	67 (50.8)	9.356 (4.803–18.224)	<.001	10.353 (5.201–20.608)	<.001	7.713 (3.330–17.867)	<.001	5.059 (2.061–12.418)	<.001
*p* For trend		3.065 (2.231–4.211)	<.001	3.248 (2.330–4.528)	<.001	2.630 (1.773–3.901)	<.001	2.147 (1.405–3.282)	<.001

*Note*: Model 1: uncorrected. Model 2: corrected for age, gender, BMI. Model 3: corrected for age, gender, BMI, subtentorial, supratentorial, COPD, myocardial infarction, atrial fibrillation, coronary heart disease, diabetes mellitus, stroke history, hypertension, hyperlipemia, heart failure, gout, current smoker, current drinker, gastric intubation, acid‐suppressing drugs. Model 4: corrected for age, gender, BMI, subtentorial, supratentorial, COPD, myocardial infarction, atrial fibrillation, coronary heart disease, diabetes mellitus, stroke history, hypertension, hyperlipemia, heart failure, gout, current smoker, current drinker, gastric intubation, acid‐suppressing drugs water swallow test, glucose, NIHSS, GCS.

### 3.4 Predictive value of SII for the development of SAP in patients with stroke

**FIGURE 1:**
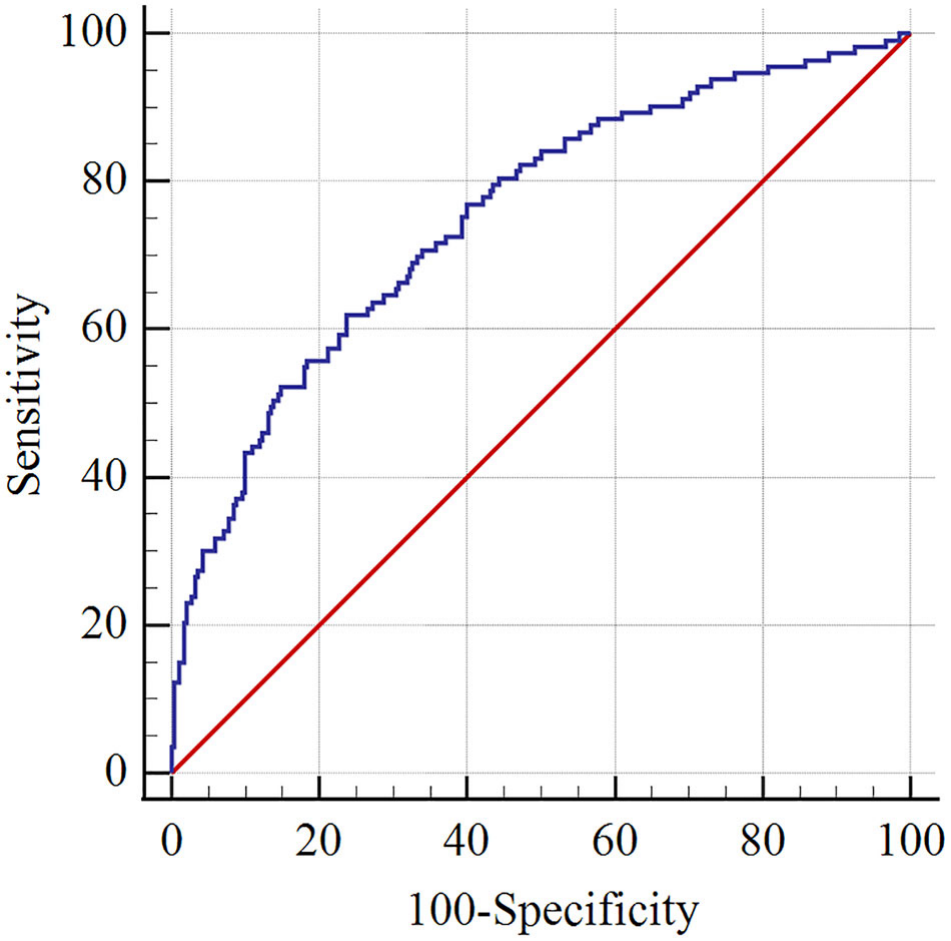
The receiver‐operating characteristic (ROC) curve was used to evaluate the diagnostic value of SII for stroke‐associated pneumonia (SAP) severity.

The ROC curve analysis showed that the area under the curve of SII predicting the occurrence of SAP in patients with stroke was 0.752 (95% CI: 0.698–0.806, *p* < .001), and the optimal cutoff value of SII predicting the occurrence of SAP was 861.01, with a sensitivity of 61.9% and a specificity of 76.2%. This suggested that SII has a certain extent of predictive value for SAP (Figure 1).

## 4 DISCUSSION

In this study, we retrospectively collected data from 395 patients with acute stroke from the First Affiliated Hospital of Kunming Medical University between January 2017 and December 2019. The results showed that SII was an independent risk factor for the development of SAP in patients with stroke, and there was a trend of a relationship between SII and the development of acute stroke, suggesting a predictive value of SII for early SAP.

SAP is a severe complication in patients with stroke and can worsen their condition, increasing morbidity and mortality. We found a 28.6% prevalence of SAP with longer hospital stay and a higher mortality rate, which is consistent with the findings of other studies (Cheng et al., 2020; Hilker et al., 2003; Smith et al., 2015). The pathogenesis of SAP in patients with stroke is unclear, but some studies suggest that immunosuppression‐induced pathological changes in the body are associated with stroke‐induced SAP (Dirnagl et al., 2007). Most studies have linked SAP to stroke‐induced immunodepression syndrome (SIDS), poststroke dysphagia, and impaired consciousness (Eltringham et al., 2018). Stroke‐induced immunodepression is a key factor in the development of SAP, leading to decreased recognition and clearance of cytotoxic T‐lymphocytes, and increased susceptibility in patients with stroke (Chamorro et al., 2012; El Husseini & Laskowitz, 2014; Meisel et al., 2005).

Patients with SAP have diverse clinical presentations and rapidly changing conditions, and most clinical indicators used to detect SAP lack specificity (Emsley & Hopkins, 2008; Finlayson et al., 2011; Li et al., 2014). The use of simple blood parameters for the early prediction of SAP has gradually gained attention in recent years. Hu et al. (2014) proposed that SII is a novel inflammatory index consisting of three cell counts: neutrophils, lymphocytes, and platelets. Many studies have shown that SII is closely related to various diseases and is a relatively good predictor of stroke prognosis (Wang et al., 2021; Wang et al., 2022; Wu, Yan et al., 2021). However, there are few studies on the relationship between SII and SAP. The results of this study may provide a theoretical basis for predicting and diagnosing SAP using SII. Considering that SII is associated with inflammatory response and immunosuppression in patients with stroke, it is possible that SIDS predicts SAP by decreasing the number of peripheral lymphocytes in the body, increasing the risk of infection in patients with stroke (Chamorro, Amaro et al., 2007; Chamorro, Urra et al., 2007). Second, in patients with stroke, an early inflammatory response occurs after brain injury. Neutrophils infiltrate and accumulate in areas of ischemic injury, secreting the inflammatory factors reactive oxygen species and matrix metalloproteinase 9, which exacerbate brain tissue damage and become a marker of poor prognosis in patients with stroke (Garau et al., 2005; Zhang et al., 2020). Endothelial cells are also damaged in patients with stroke, leading to platelet activation, the expression of adhesion molecules, and the release of inflammatory factors, resulting in atherosclerosis, thrombosis, and a decrease in platelet count (McMorran et al., 2009; Modjeski & Morrell, 2014). In addition, it has been shown that T‐lymphocytes and neutrophil‐to‐lymphocyte ratio have been shown to predict SAP development (Feng et al., 2018; Wu, Zhang et al., 2021). However, SII provides a more stable and objective response to changes in neutrophil, lymphocyte, and platelet counts in patients with stroke and can be used to predict SAP. Our study revealed that the SII level was higher in the SAP group than in the non‐SAP group (*p* < .05), and higher SII was a risk factor for SAP in patients with stroke, whereas the results of ROC curve analysis showed that SII had a predictive value for SAP.

This study has some limitations: First, it is a single‐center retrospective study with possible selection bias and information bias, and other inflammatory markers associated with SAP, such as calcitonin, c‐reactive protein, were not collected for comparative studies with SII. Further validation with a large multicenter sample is needed in the future. In conclusion, our study showed that a high SII was an independent risk factor for the development of SAP in patients with stroke and had some predictive value for the development of SAP.

## AUTHOR CONTRIBUTIONS

Zhanhang Cui, Sai Kuang, Xiaorong Yang, Ying Wang, Shanshan Gu, Hongyu Li, and Huibin Chen contributed to the study conception and design. Sai Kuang was responsible for the design methodology. Xiaorong Yang and Ying Wang were responsible for material preparation. Shanshan Gu, Huibin Chen, and Hongyu Li were responsible for data collection. Zhanhang Cui and Sai Kuang were responsible for data analysis. Haimei Sun and Yanbing Han were responsible for the preparation of the first draft and review. All authors read and approved the final manuscript, and Haimei Sun and Yanbing Han confirmed the authenticity of all original data.

## CONFLICT OF INTEREST STATEMENT

The authors declare that there are no conflicts of interest associated with this study.

### PEER REVIEW

The peer review history for this article is available at https://publons.com/publon/10.1002/brb3.3302.

## Data Availability

The raw data supporting the conclusions of this manuscript will be made available by Haimei Sun (13769186480@139.com), without undue reservation, to any qualified researcher.
